# The ATIPAN project: a community-based digital health strategy toward UHC

**DOI:** 10.1093/oodh/oqae011

**Published:** 2024-02-20

**Authors:** Pia Regina Fatima C Zamora, Jimuel Celeste, Roselle Leah Rivera, John Paul Petrola, Raphael Nelo Aguila, Jake Ledesma, Miles Kaye Ermoso, Romulo de Castro

**Affiliations:** Center for Informatics, University of San Agustin, General Luna Street, Iloilo City 5000, Philippines; Center for Informatics, University of San Agustin, General Luna Street, Iloilo City 5000, Philippines; Department of Women and Development Studies, College of Social Work and Community Development, University of the Philippines Diliman, Magsaysay Avenue, Diliman, Quezon City 1101, Philippines; Center for Heritage and Indigenous Cultures, University of San Agustin, General Luna Street, Iloilo City 5000, Philippines; Center for Informatics, University of San Agustin, General Luna Street, Iloilo City 5000, Philippines; Center for Heritage and Indigenous Cultures, University of San Agustin, General Luna Street, Iloilo City 5000, Philippines; Center for Heritage and Indigenous Cultures, University of San Agustin, General Luna Street, Iloilo City 5000, Philippines; Center for Informatics, University of San Agustin, General Luna Street, Iloilo City 5000, Philippines

**Keywords:** Telehealth, Telemedicine, Universal Health Care (UHC), Geographically Isolated and Disadvantaged Area (GIDA), Healthcare Coordinator (HC), Electronic Medical Record (EMR)

## Abstract

The ATIPAN Project is a digital strategy aimed toward providing health services to marginalized and vulnerable communities in Western Visayas, Philippines. This paper presents the implementation of its telemedicine component in 10 partner communities, output and potential utilization in realizing Universal Health Care (UHC), and moving-forward strategies for sustainability. It also describes the hindrances and corresponding solutions identified during the 2-year project implementation. While regional in nature, the adoption of the ATIPAN model for the UHC implementation all over the Philippines could ensure health care delivery in marginalized and underserved areas.

## INTRODUCTION

In the Philippines, the Republic Act No. 11223, otherwise known as the Universal Healthcare Act (UHC), declares the ‘policy of the State to protect and promote the right to health of ALL Filipinos and instill health consciousness among them’ [1]. Section 5 of the Implementing Rules and Regulations (IRR) of RA 11223 further states that ‘Every Filipino citizen shall be automatically included into the National Health Insurance Program’ [2]. This law enables health care delivery for all Filipinos, specifically those still unreached by our health care system.

In the advent of the UHC rollout in the Philippines, there are still persisting health and social inequities in remote communities that need to be addressed for the implementation to succeed [3]. Effective and timely health care delivery is affected by complex factors including distance to the nearest health facility, terrain that hinders residents and health care workers from traveling, armed conflict, cultural and language barriers and lack of financial resources to prioritize medical needs [4]. Moreover, the barangay health workers (BHWs), whose role is to carry out health profiling to ensure that health needs are documented and met, may not be able to fulfill their roles due to numerous factors. Lived realities, such as the inadequate number of BHWs in an area, delayed honorarium and their informal role as contingency workforce of nearby understaffed health centers are identified factors affecting their performance [5].

A possible way to circumvent these inequities and enable health care delivery in remote areas is implementing telehealth strategies that involve community-based trained patient navigators and digital infrastructure capacitation [6, 7]. The ATIPAN Project was designed to provide telehealth services to 10 underserved and under-resourced partner communities in Western Visayas, Philippines as a response to the disproportionate effects of the COVID-19 pandemic in these communities, including the lacking or limited health care access affecting the management of other disease conditions among the residents [8]. This manuscript highlights ATIPAN as a digital health strategy toward the prioritization of these communities and a means to efficiently achieve UHC in the Philippines. The discussion will focus primarily on the telemedicine aspect of the project.

## MATERIALS AND METHODS

Inspired by the Hiligaynon word *atipan* which means ‘to take care of’, the ATIPAN Project was implemented in November 2021 to provide free teleconsultations, technology and training for community health coordinators (HCs) and basic medication and health supplies [8].

### Partner communities

Our partners are 7 *Ati* Indigenous People (IP) communities and 3 Rural Low Income (RLI) communities. The estimated total population of all partner communities is 5646 individuals. Table 1 shows the profile of the communities.

**Table 1:** Community Profiles

**Community**	**IP/RLI**	**Estimated Population**
Malay Ati Tribal Association (MATA) Community	IP	900
Kabangrusan Ati Community	IP	1086
Mt. Tag-ao Ati Community	IP	535
Dacal Ati Community	IP	425
Pantad Ati Community	IP	105
Lanit Ati Village	IP	140
Kati-Kati Ati Community	IP	405
Center for Agrarian Reform and Rural Development (CARRD) - assisted agrarian reform communities (Sto.Tomas and Salngan, Passi)	RLI	600
Typhoon Yolanda Pabahay (Bulaquena, Gogo, Calapdan, and Tacbuyan, Estancia)	RLI	1000
Migrants Community (Capoyuan and Poblacion, Barbaza)	RLI	450

**Figure 1:**
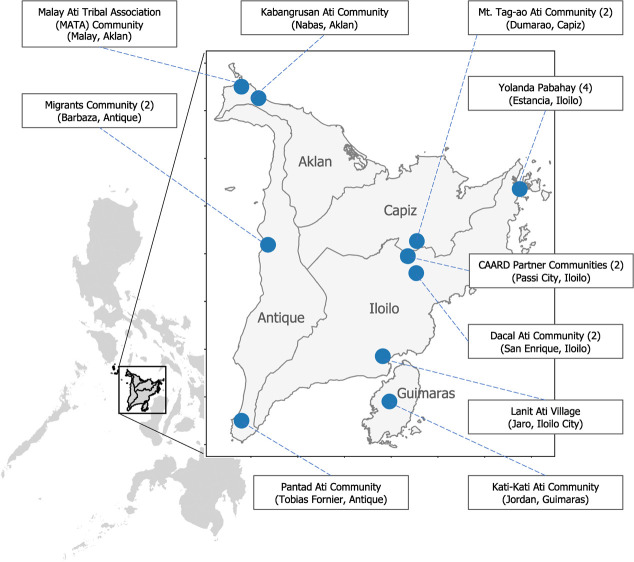
Locations of the partner communities in Western Visayas (region 6), Philippines

Based on socio-economic factors and remoteness from the nearest health facility, seven (7) communities are categorized as Geographically Isolated and Disadvantaged Area (GIDA), defined as ‘far-flung areas and marginalized populations which include islands, mountainous areas, conflict-affected areas (CAAs), internally displaced persons (IDPs) and indigenous cultural community or indigenous people (ICCs/IPs)’ [9, 10]. Our communities are mostly located in agricultural and coastal areas in Panay and Guimaras islands. One (1) community is in an urban relocation site. Figure 1 shows the locations of the partner communities.

### Project implementation

**Figure 2:**
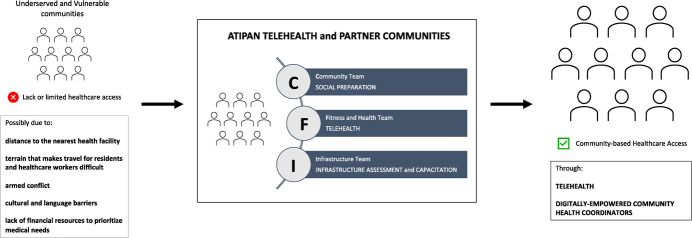
Addressing limited health care access through Telehealth.

The implementation of ATIPAN involved 3 phases: 1) Social Preparation, 2) Infrastructure Assessment and Capacitation, 3) Telemedicine. Three (3) teams were formed to implement these phases: Community (C) Team, Fitness and Health (F) Team, Infrastructure (I) Team. Figure 2 shows the identified limitation of health care access in underserved and vulnerable communities in the Philippines and how the ATIPAN Telehealth project addressed it.

1) Social Preparation: This phase was implemented by our C team composed of an IP Community Coordinator, a RLI Community Coordinator and community assistants. Community partnerships were premised on the following:

a) Partnership engagement with the community coordinators of at least 5 years,
b) Availability of electricity and internet signal and
c) Willingness to participate as a partner community.

The social preparation for the 10 communities were rooted in ethnographic approaches, *paghinun-anon* (dialogue) and appreciative inquiry [11, 12]. For the IP communities, their social preparation was also based on the Indigenous Peoples’ Rights Act of 1997 and the Indigenous Knowledge Systems and Practices (IKSPs) and Customary Laws (CLs) Research and Documentation Guidelines of 2012 of the National Commission on Indigenous Peoples (NCIP) [13, 14]. The basic documents that were processed for each community were the Resolution of Consent, a Memorandum of Agreement between the implementing agency (Center for Informatics) and each community and the Certificate of Precognition from the NCIP.

Dialogue and appreciative inquiry included meetings and disclosure conferences conducted from July 2021 to March 2022. During these sessions, the communities’ choice of health coordinator/s was also discussed. The criteria for selecting HCs were:

a) Community knowledge and trust
b) Literacy
c) Familiarity with the use of mobile devices (at least a smartphone)
d) Residing in an area of the community with a reliable (or available) internet signal

One (1) health coordinator (HC) was assigned to every 300 individuals (or less) in a community. In total, twenty-three (23) HCs were selected to facilitate telehealth in the partner communities.

For the entire project duration, telehealth implementation in partner communities were also monitored based on monthly registrations and consultations, access to medications and diagnostic tests, referral to medical services beyond telehealth and overall assessment of the intervention in the communities. Monitoring was done through direct interviews, community meetings, workshops and informal inquiries via messaging platforms.

2) Infrastructure Assessment and Capacitation: This phase was implemented by our I team composed of a software developer and a data analyst. ATIPAN partner communities are in remote areas where there is poor internet connection which may affect the quality of videoconferencing during telehealth [15]. As such, a qualitative assessment of the available internet connectivity options and appropriate video conferencing platforms for teleconsultations was done in each community. A scoring system was used to assess different online platforms while using available internet sources in each community. The parameters included were as follows:
1) **Video Clarity (VCL)**: 5 (video is clear, details are sharp), 1 (video is highly pixelated)
2) **Video Consistency (VCS)**: 5 (video is continuous, minimal video interruptions), 1 (video is frozen most of the time)
3) **Audio Clarity (ACL)**: 5 (audio is clear and audible), 1 (audio is inaudible)
4) **Audio Consistency (ACS)**: 5 (audio is continuous, minimal audio interruptions), 1 (audio breaks)
5) **Video and Audio Latency (VAL)**: 5 (video and audio are synced, minimal call latency between call endpoints), 1 (video and audio are delayed, high latency between call endpoints).

Signal and platform assessments and recommendations for each community are shown in Table 2. Other details on the said assessments are in Supplementary Material 1.

**Table 2:** Recommended Network Providers and Communication Platforms for ATIPAN Telemedicine

**Community/Site**	**Philippine Network Providers**						**Video Conferencing Platform Assessment Scores** ^**a**^					
	**Smart**	**TNT**	**Globe**	**TM**	**DITO**	**PLDT**	**Jitsi**	**Messenger**	**Viber**	**Telegram**	**Zoom**	**Skype**
Pantad Ati Community				✓				17				
Kati-Kati Ati Community						✓		22				
Mt. Tag-ao Ati Community					✓		20					
Dacal Ati Community					✓						22	
CAARD (Sto. Tomas)			*PisoNet*					16				
CAARD (Salngan)	*Boosted*							21				
MATA Community					✓							21
Kabangrusan Ati Community					✓							22
Migrants (Capoyuan)				✓					19			
Migrants (Poblacion)			✓					21				
Yolanda Pabahay (Gogo)	✓							20				
Yolanda Pabahay (Tacbuyan)	✓											22
Yolanda Pabahay (Bulaqueña)	✓							22				
Yolanda Pabahay (Calapdan)	✓											23
Lanit Ati Village						✓			22			

a The highest possible score is 25 (for the best quality).

All HCs were provided with mobile phones and tablets pre-configured with the relevant software for performing telehealth consultations. Other mobile device accessories such as phone and tablet screen protectors, cases, earphones and power banks were also provided. Subscriber Identity Module (SIM) cards of the selected telecommunication network providers for each community were distributed. The HCs were also supplied with data credits for calls, texts and internet on a monthly plan.

Finally, UnEMR (the Uncomplicated Electronic Medical Record), which was developed originally for doctors only, was retooled in another instance to include a portal for the HCs to register patients [16]. The retooled UnEMR became UnEMR Tele. Features that allowed scheduling teleconsultations as well as an integration with a video conferencing platform, Jitsi, were incorporated [17].

3) Telemedicine
  The last phase was implemented by the F team. It is led by a telehealth coordinator, who oversees teleconsultation services, patient referrals, medication supply and availability of requested laboratory tests and imaging. It also includes doctors from different specializations who mainly conduct the teleconsultations. Majority of the doctors are women. Table 3 shows the specializations and gender of ATIPAN doctors.
  The doctors and HCs underwent 2 workshops on Health Data Ethics, Cultural Sensitivity (for doctors), Body and Boundaries (for HCs) and the use of the mobile devices and UnEMR Tele. The HCs were also trained to use medical equipment for vital signs and body mass index (BMI) measurements. Prescribed medications and/or laboratory tests were made available through the project and the local municipal or rural health units (MHUs or RHUs).
  ATIPAN teleconsultation services started in June 2022. At the community level, the health care delivery team is composed of the doctors (online) and the HCs. The location of teleconsultations varies across the communities. It can be in a designated area within the community, a patient’s home, or the HC’s home. The workflow for conducting a teleconsultation is as follows:
1) An HC registers the patient in UnEMR tele. A teleconsultation is scheduled by the HC based on the patient or guardian’s availability.
2) On the day of the consultation, the doctor starts a video conference through the recommended platform. If a video conference or video call is not possible, the consultation is performed through a phone call instead. If the teleconsultation still fails, it is rescheduled. During the teleconsultation, the doctor encodes patient encounter data in UnEMR Tele.
3) After a consultation, the doctor sends the patient's prescriptions and laboratory and imaging orders to the HC via the latter's preferred platform (text, email, Facebook Messenger, etc).
4) The HC sends the prescriptions and orders online, or provides print copies to the patient (or guardian and family members). For referrals to other health facilities, patients are guided by the telehealth coordinator and the HC.

**Table 3:** Medical Specializations of ATIPAN Doctors

**Specialization**	**Female**	**Male**	**Total**
General practice	5	1	6
Internal medicine	2	1	3
Pediatrics	3	1	4
Psychiatry	1	0	1
Orthopedics	0	1	1
Obstetrics and gynecology	1	0	1
Family and community medicine^a^	1	0	1
TOTAL	13	4	17

a The FCM doctor is affiliated with the National Commission on Indigenous Peoples (NCIP), and assigned to IPs in 3 regions: Western Visayas, Central Visayas, Eastern Visayas.

At times, the internet signal fails when there is a large influx of patients during clinic hours. In these situations, patient registration and consultations are conducted *ad hoc* by telephone, and paper recording ensues. The telehealth coordinator, with the help of the HCs and doctors’ notes, belatedly encodes patient and encounter details.

**Figure 3:**
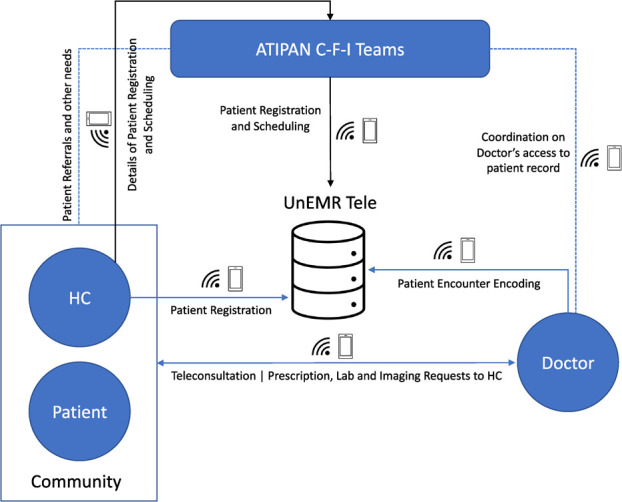
Workflow of ATIPAN Teleconsultations Vis-a-Vis the telehealth platform (UnEMR Tele).

The current workflow of ATIPAN teleconsultations is represented in Fig. 3.

Entries in the UnEMR Tele are analyzed monthly to generate community health data-aggregate data on patient registrations, consultations, diagnoses and prescriptions.

## RESULTS AND DISCUSSION

### Atipan outputs and potential for UHC

The Philippine UHC law aims to empower local health units to provide primary health care services [2]. However, easy and timely access even to these facilities is still a challenge for communities living in remote areas. In 2020, only 50% of Filipinos were able to access primary health care services within 30 minutes [18]. This barrier leads to poor health outcomes that are entirely preventable with better and more equitable access [19].

### Health care access

**Figure 4:**
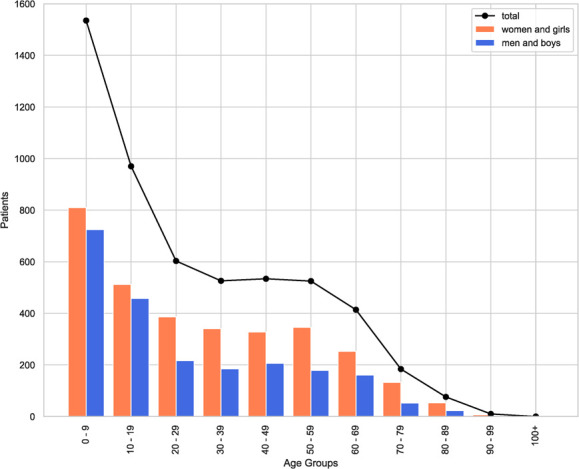
Sex and age distribution of patients registered in the ATIPAN–UnEMR Tele database

**Figure 5:**
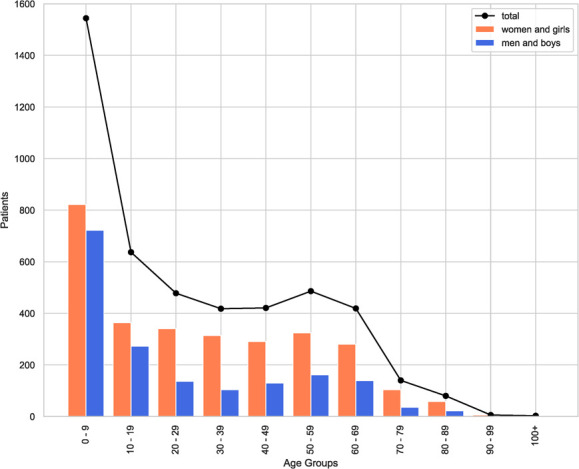
Sex and age distribution of teleconsultation patients in the ATIPAN–UnEMR Tele database

In partner communities, ATIPAN has served as a patient’s access point to health care. From May 2022 to July 2023, 5691 people from the partner communities and surrounding areas were registered in UnEMR Tele. From the pool of registered patients, 4874 initial and follow-up teleconsultations were conducted. Majority of the patients seen were of pediatric age, followed by the older age groups. There are also more women and girls than men and boys consulting. Figures 4 and 5 show the sex and age distribution of patient registration and consultation from May 2022 – July 2023.

The availability of health services through ATIPAN gained favor among community residents. In interviews with the HC from the Kati-Kati Ati community [20] and the tribal leader of Pantad Ati Community [21], they stated the benefits of telehealth to their community:

*‘...ang ATIPAN Project gin disenyo para idul-ong sa amon ang serbisyo nga amon kinahanglan.’*

(‘..the ATIPAN project was designed to bring medical services that [the community] needs.’)

*‘...[Ang proyekto] importante ilabi nagid kung may mga balatian kag malayo ang ospital..mas ayo gid nga may telehealth diri sa community mismo para hindi ka na magastusan pa sang pamasahe.’*

(‘..[ATIPAN] is important especially if there are diseases/conditions [among residents] and we still need to travel far to get to a hospital…it is good that there is telehealth in the community so that we no longer have to pay for travel.’)

Similar testaments have also been narrated by other communities indicating a potential for ATIPAN and similar programs to transform the landscape of health care delivery in remote and under-resourced communities, primarily by providing access to health services within, thereby relieving some financial burdens associated with health care (i.e. transportation). While the long-term benefits of ATIPAN in these communities have not yet been fully assessed, we see evidence of its potential from other telemedicine/telehealth efforts in other parts of the world. For example, the Alaska Federal Health Care Access Network (AFHCAN) catering to Alaskan Natives launched its telehealth solution as far back as 2001. Their store-and-forward telehealth approach improved health care access and reduced the cost of health care delivery [22].

Moreover, while the ATIPAN model started only with telehealth consultations, it has already laid the foundations for a well-rooted Primary Health Care Provider Network in partner communities located in GIDAs [23, 24]. The ATIPAN network currently has 2 very effective components: 1) manpower resources trained in the use of digital health tools – the telehealth doctors and the community-based HCs (patient navigators for their community members), 2) infrastructure resources – the UnEMR Tele platform, internet connectivity, mobile devices for telehealth which were thoroughly prepared ahead of implementation and fully supported by our I team. Adopting a digital health intervention like ATIPAN could facilitate the Department of Health (DOH) and local government units (LGUs) to fulfill their mandate to enabling a health care delivery system that will give Filipinos access to a primary care provider (UHC IRR Section 6.6.4) [2] especially in isolated, remote and under-resourced areas.

Our work on ATIPAN has shown that community-empowered telehealth services is a way of providing health care services to marginalized, vulnerable and remote communities in the Philippines. Decades of development program experiences have showcased how participatory approaches are vital in engaging with communities [25, 26]. In the ATIPAN case, conversations and discussions with leaders were important for communities to understand, question, clarify and negotiate before community resolutions were issued to signify their agreement on the design and processes related to project collaboration. It also became integrative as it created a space for caring within and outside the community. One of the IP partner communities has already started sharing ATIPAN teleconsultation services to residents from surrounding communities. Residents from these disempowered communities now have the means to promote people’s well-being through digital health tools. Furthermore, the partner communities also demonstrated community ownership of ATIPAN through innovations that were entirely their own: 1) strengthening internet signal with an antenna extension, 2) conducting house-to-house patient finding missions to catch elderly and non-ambulatory individuals and 3) nutritional/feeding community gatherings to register patients. Truly, engaging the communities through this approach will pave the way to sustainable utilization of digital health strategies that is vital to achieving UHC in the Philippines.

### Health data for UHC

Another important contribution of ATIPAN is the generation of health data specifically fit for UHC rollout in GIDAs. An analysis of UnEMR Tele records provides information on the demographic profile of patients from the communities and clinical data of patients. As of July 31, 2023, the Top 15 diagnoses or conditions of patients included both communicable and non-communicable diseases. The top prescribed medications, consisting of medications for symptomatic relief, antibiotics and anti-hypertensive medications, are consistent with the prevalent conditions. This information is shown in Tables 4 and 5.

**Table 4:** Top 15 diagnoses or conditions of ATIPAN patients (as of July 31, 2023)

**Diagnosis/Condition**	**Frequency**
Upper respiratory tract infection	767
Community-acquired pneumonia	270
Hypertension	238
Pediatric community-acquired pneumonia	202
Hypertensive and cardiovascular disease	168
Well child	131
Urinary tract infection	115
Bronchial asthma	111
Benign paroxysmal positional vertigo	83
Osteoarthritis	74
Non-ulcerative dyspepsia	77
Tuberculosis	67
Malnutrition	85
Hypersensitivity reaction	58
Diabetes mellitus type 2	62

**Table 5:** Top 15 prescribed medications for ATIPAN patients (as of July 31, 2023)

**Prescribed medication**	**Frequency**
Multivitamins	2965
Cetirizine	1074
Carbocisteine	365
Paracetamol	360
Salbutamol	339
Co-amoxiclav	293
Losartan	245
Amlodipine	213
Amoxicillin	215
Mefenamic acid	174
Betahistine dihydrochloride	141
Cefalexin/cephalexin	141
Omeprazole	123
Celecoxib	119
Diphenhydramine	128

Utilization of health data from electronic medical records (EMR) was shown to improve health outcomes, health policies and health care delivery as a whole [24, 27]. This has been observed in the Indian Health Service Joslin Vision Network Tele-Ophthalmology (IHS JVN) Program where virtual diabetic retinopathy surveillance among American Indians and Alaska Natives is conducted. This has increased compliance among diabetic patients and enabled timely management to prevent vision loss [28]. Similar to the IHS JVN Program, data from ATIPAN also has the potential to improve health care in GIDA communities by its use to model the health status of specific communities based on illnesses observed and estimate the actual cost of health care based on diagnoses, prescriptions and laboratory tests ordered. Modeling healthcare costs with highly focused community data will produce more accurate resources allocation by the respective local governments. In fact, the National Commission on Indigenous People (NCIP, Regions 6, 7, 8) has already requested for ATIPAN data to guide their procurement of medications. In addition, health monitoring agencies, such as the DOH, can also utilize ATIPAN data in monitoring the effectiveness of their health programs for malnutrition [29], tuberculosis [30], other infectious diseases [31, 32] and lifestyle-related conditions [33] that are prevalent in these communities.

## ISSUES IN TELEHEALTH IMPLEMENTATION

### Infrastructure

At present, the main challenge during teleconsultations is still the fluctuations of the internet signal strength, specifically during inclement weather. Phone calls are utilized as a last resort when the internet signal strength cannot accommodate video conferencing. Over time, we also observed that the practice of the HCs shifted to the use of Facebook Messenger as their main telehealth platform. According to them, while other platforms have better video and audio quality in the initial assessment, teleconsultations push through even during bad weather when Facebook Messenger is used. Thus, there may be a need to reassess the digital platforms recommended to the communities.

### Telemedicine

Here are observations and innovations in the conduct of telemedicine in partner communities:

1) Teleconsultations that are scheduled in advance are not necessarily kept. At times, the patient and/or guardian of children prioritize family commitments and work opportunities. This demonstrates that economic needs take precedence over health care, which is not unexpected. To address this, we have instituted *telehealth doctor clinic hours* for walk-ins.
2) Non-ambulatory patients also benefit from telehealth. Our HCs visit patients’ homes and facilitate the teleconsultation there. When it cannot be performed because of the lack of an *onsite* internet signal, HCs write down notes on the patients’ conditions and relay them to the doctor who gives instructions that are relayed back to the patients or a guardian. For all cases where the information given is not substantial to generate an appropriate medical management, the patients and/or guardian are advised referral to the nearest rural health unit or clinic.
3) The unavailability of free medicine discourages health-seeking behavior. Even if the teleconsultations are free, patients may still forgo them if medications are not dispensed for free afterward. Patients in our communities often do not have the means to buy their own medicines. Thus, their health decisions come in conflict with their economic situation. This observation emphasizes that health programs cannot be independent of economic upliftment programs since health care is still viewed by many Filipinos as a cost rather than a right.
4) Patient registration is a leading measure followed closely by teleconsultations. Prior to the teleconsultations in June 2022, we started a month ahead in pre-registering patients simply to populate our telehealth database. We were surprised to find that the subsequent consultations that followed mirrored patient registration by volume. This means that if we register patients ahead, they are likely to utilize telehealth.
5) While the ATIPAN model was able to provide teleconsultation services and generated health data from partner communities, its utilization in the roll-out of UHC in GIDAs can be made sustainable by 1) institutionalizing the role of local HCs in marginalized and vulnerable communities and 2) engaging the local government unit (LGU) in establishing a reliable referral network (provision of medications, laboratory testing, imaging and transport of indigent patients to and from appropriate health facilities).

## CONCLUSION

The ATIPAN Project is proof of concept that a digital health strategy, which engages the community, enables health care delivery in marginalized and vulnerable areas in the Philippines. More than laying down foundations for primary health care access, the ATIPAN model also generated data with potential uses to guide public health programs in efficient health care delivery and resources planning, and as a health baseline for monitoring diseases in the communities moving forward. To sustain telehealth efforts, issues such as fluctuating internet signal and lack of a reliable referral network for prescribed medications, laboratory tests and imaging need to be addressed. Overall, utilizing the ATIPAN model will benefit UHC implementation in the country as it paves the way for remote areas to have health care access.

## Supplementary Material

Web_Material_oqae011

## Data Availability

Health data presented in this article were generated from patient teleconsultations done in the partner communities. Data will be shared upon request to the corresponding author with grant of permission of the partner communities and approval of the National Commission on Indigenous Peoples (NCIP) of the Philippines.
