# Correction to: The ATIPAN project: a community-based digital health strategy toward UHC

**DOI:** 10.1093/oodh/oqae024

**Published:** 2024-08-05

This is a correction to: Pia Regina Fatima C Zamora, Jimuel Celeste Jr., Roselle Leah Rivera, John Paul Petrola, Raphael Nelo Aguila, Jake Ledesma, Miles Kaye Ermoso, Romulo de Castro, The ATIPAN project: a community-based digital health strategy toward UHC, *Oxford Open Digital Health*, Volume 2, 2024, oqae011, https://doi.org/10.1093/oodh/oqae011

In the originally published version of this manuscript, the second-listed author's name was given as Jimuel Celeste, instead of Jimuel Celeste, Jr.

In Table 2, the highlights indicating Recommended Network Providers and Communication Platforms for ATIPAN Telemedicine for each Community/Site were erroneously omitted.

Errors were present in the numbering of references 15-23 and their in-text citations.

The manuscript has been corrected.

