# Supplementary material for: The ATIPAN project: a community-based digital health strategy toward UHC
Source: Oxf Open Digit Health. 2024 Feb 20;2:oqae011. doi: 10.1093/oodh/oqae011 (PMC11932401; doi:10.1093/oodh/oqae011)
Supplement: Web_Material_oqae011 [file Web_Material_oqae011.pdf]

Community	Date of Testing	Networks Available	Download Speed (mbps)	Upload Speed (mbps)	Network Tested against Video Conferencing Platforms (based on availability and speed)	Platforms Tested	Video Clarity (VCL)	Video Consistency (VCS)	Audio Clarity (ACL)	Audio Consistency (ACS)	Video Audio Latency (VAL)	Total	Recommendations	Other Comments
Pantad Ati Community, Igcalawagan, Tobias Fornier, Antique	19 Jan 2022	TM	1	3.71	TM	FB Messenger	3	3	4	4	3	17	Network: TM<br>Primary Platform: FB Messenger<br>Alternatives: Jitsi	
						Viber	2	3	4	3	4	16		
						Zoom	3	2	4	3	3	15		
						Jitsi	4	2	4	3	4	17		
Kati-Kati Ati Community, San Miguel, Jordan, Guimaras	21 Jan 2022	TNT			PLDT	FB Messenger	4	5	4	4	5	22	Network: PLDT<br>Primary Platform: FB Messenger<br>Alternatives: Viber	- Jitsi may be used as the primary platform if the video is off during the consultation. If video is needed, Skype may be used for the consultation instead.
		PLDT	1.55	1.76		Viber	3	4	5	4	4	20		
						Skype	4	3	4	4	4	19		
						Zoom	3	2	4	3	3	15		
						Jitsi	4	2	4	3	3	16		
Sitio Tag-ao, Tamulalod, Dumarao, Capiz	24 Jan 2022	Globe	0.82	0.01	DITO	FB Messenger	3	3	4	3	4	17	Network: DITO<br>Primary Platform: Jitsi<br>Alternatives: Skype	- Since a PLDT broadband connection is available for this community, we can look into installing a line for them sometime in the future.
		DITO	14.7	0.09		Viber	3	2	3	3	2	13		
		PLDT	1.97			Skype	4	4	4	4	4	20		
						Zoom	3	1	4	3	3	14		
						Jitsi	4	4	4	4	4	20		
Dacal Ati Community, Lip-ac, Abaca, Iloilo	24 Jan 2022	Smart	5.75	7.48	DITO	FB Messenger	4	3	4	4	4	19	Network: DITO<br>Primary Platform: Zoom<br>Alternatives: FB Messenger/Skype/Jitsi	- Community has a good and stable connection through DITO. Any of the platforms can be used for tele consultations; however, Skype and Zoom had the highest quality among all of them.
		DITO	18.5	8.14		Viber	3	3	4	4	4	18		
						Skype	4	3	4	4	4	19		
						Zoom	5	4	5	4	4	22		
						Jitsi	3	4	4	4	4	19		
CAARD (Community A), Sto. Tomas, Passi, Iloilo	31 Jan 2022	Globe - PisoNet	1.44	0.99	Globe - PisoNet	FB Messenger	4	3	4	3	2	16	Network: Globe - PisoNet<br>Primary Platform: FB Messenger<br>Alternatives: Skype	- Mobile network signals are not available for this community.<br>- The community has a public WIFI installed (PisoNet), with rates of P20/3hrs. We might be able to use this while we look into other internet options for them.
						Skype	4	3	4	3	2	16		
						Zoom	4	3	4	2	2	15		
						Jitsi	3	2	3	2	1	11		
CAARD (Community B), Salngan, Passi, Iloilo	31 Jan 2022	Smart - with booster			Smart - with booster	Messenger	5	4	4	4	4	21	Network: Smart<br>Primary Platform: Messenger<br>Alternatives: None	- Need to utilize mobile network boosters to improve internet connectivity<br>- Was only able to test Messenger during testing
Malay Ati Tribal Association, Cubay Sur, Malay, Aklan	1 Feb 2022	Globe	3.06	0.03	Globe	FB Messenger	3	3	4	3	2	15	Network: DITO<br>Primary Platform: Skype<br>Alternatives: Zoom	- A Globe tower is being built around the area so once that finishes, Globe might become the best network to use for this community.
						Viber	3	3	4	3	3	16		
						Skype	4	3	4	3	3	17		
						Zoom	3	1	4	3	4	15		
						Jitsi	2	1	3	2	1	9		
		DITO	5.06	2.91	DITO	FB Messenger	4	3	4	3	3	17		
						Viber	4	4	4	4	4	20		
						Skype	5	4	4	4	4	21		
						Zoom	5	4	4	4	4	21		
						Jitsi	3	4	4	3	4	18		
Kabangrusan Ati Community, Butong Laserna, Nabas, Aklan	1 Feb 2022	Smart (4.77 / 0.02)	4.77	0.02	DITO	FB Messenger	3	3	3	3	3	15	Network: DITO<br>Primary Platform: Skype<br>Alternatives: Jitsi	
		Globe (12.1 / 0.36)	12.1	0.36		Viber	3	4	4	4	4	19		
		DITO (53.3 / 12.3)	53.3	12.3		Skype	5	4	4	4	5	22		
						Zoom	4	4	4	4	5	21		
						Jitsi	4	4	4	4	4	20		
Barbaza Migrant Families A, Barbaza, Antique	2 Feb 2022	TM (11.1 / 1.50)	11.1	1.5	TM	Viber	3	4	4	4	4	19	Network: TM<br>Primary Platform: Viber<br>Alternatives: Messenger (PisoNet)	- Although TM showed good speed during the speed test, we were unable to have a stable video conference using it.<br>- However, we might still use TM as the alternative network for this community.<br>- According to the community, PLDT broadband line installation is possible.
		PisoNet (0.02 / 0.01)	0.02	0.01	PisoNet	FB Messenger	4	4	4	3	3	18		
						Viber	3	4	4	3	4	18		
						Skype	3	4	4	3	4	18		
						Zoom	4	3	4	3	4	18		
						Jitsi	3	4	3	3	4	17		
Barbaza Migrant Families B, Barbaza, Antique	2 Feb 2022	Globe (11.6 / 0.31)	11.6	0.31	Globe	FB Messenger	4	4	4	4	5	21	Network: Globe<br>Primary Platform: FB Messenger<br>Alternatives: Viber/Skype	
						Viber	3	4	4	4	5	20		
						Skype	5	3	4	4	4	20		
						Zoom	5	3	4	3	4	19		
						Jitsi	4	3	4	3	4	18		
Gogo, Estancia, Iloilo (Typhoon Yolanda Pabahay)	3 Feb 2022	Smart (18.5 / 24.4)	18.5	24.4	Smart	FB Messenger	4	4	4	4	4	20	Network: Smart<br>Primary Platform: FB Messenger<br>Alternatives: Skype/Zoom/Jitsi	
		TM (4.22 / 0.31)	4.22	0.31		Viber	3	4	4	4	4	19		
						Skype	5	3	4	4	4	20		
						Zoom	4	4	4	4	4	20		
						Jitsi	4	4	4	4	4	20		
Tacbuyan, Estancia, Iloilo (Typhoon Yolanda Pabahay)	3 Feb 2022	Smart (7.27 / 24.5)	7.27	24.5	Smart	FB Messenger	4	4	4	4	4	20	Network: Smart<br>Primary Platform: Skype<br>Alternative: Zoom	
						Viber	4	4	4	4	4	20		
						Skype	5	5	4	4	4	22		
						Zoom	4	5	4	4	5	22		
						Jitsi	4	4	4	4	5	21		
Bulaquena, Estancia, Iloilo (Typhoon Yolanda Pabahay)	3 Feb 2022	Smart (3.47 / 27.0)	3.47	27	Smart	FB Messenger	4	4	5	4	5	22	Network: Smart<br>Primary Platform: FB Messenger<br>Alternative: Zoom/Jitsi	- PLDT broadband line installation is possible.
						Viber	5	4	4	4	3	20		
						Skype	4	4	4	4	4	20		
						Zoom	5	4	4	4	4	21		
						Jitsi	4	4	4	4	5	21		
Calapdan, Estancia, Iloilo (Typhoon Yolanda Pabahay)	3 Feb 2022	Smart (31.0 / 1.28)	31	1.28	Smart	FB Messenger	4	3	4	4	4	19	Network: Smart<br>Primary Platform: Skype<br>Alternative: Zoom/Jitsi	
		Globe (2.71 / 0.26)	2.71	0.26		Viber	4	3	4	4	4	19		
						Skype	5	4	5	4	5	23		
						Zoom	4	4	4	4	4	20		
						Jitsi	4	4	4	5	3	20		
Ati Village, Lanit, Jaro, Iloilo City	18 Feb 2022	PLDT (21.2 / 22.4)	21.2	22.4	PLDT	FB Messenger	4	4	4	4	5	21	Network: PLDT<br>Primary Platform: Viber<br>Alternative: FB Messenger/Skype/Jitsi	
						Viber	5	4	4	4	5	22		
						Skype	4	4	4	4	5	21		
						Zoom	4	3	4	4	4	19		
						Jitsi	4	4	4	4	5	21		
